# The impact of patient-reported outcome data from clinical trials: perspectives from international stakeholders

**DOI:** 10.1186/s41687-020-00219-4

**Published:** 2020-07-02

**Authors:** Samantha Cruz Rivera, Christel McMullan, Laura Jones, Derek Kyte, Anita Slade, Melanie Calvert

**Affiliations:** 1 Centre for Patient Reported Outcomes Research, Institute of Applied Health Research, College of Medical and Dental Sciences, University of Birmingham, Birmingham, UK; 2 Institute of Applied Health Research, College of Medical and Dental Sciences, University of Birmingham, Birmingham, UK; 3 National Institute for Health Research (NIHR) Birmingham Biomedical Research Centre, University of Birmingham, Birmingham, UK; 4 NIHR Surgical Reconstruction and Microbiology Research Centre University Hospitals Birmingham NHS Foundation Trust and University of Birmingham, Birmingham, UK; 5 National Institute for Health Research (NIHR) Applied Research Centre West Midlands, University of Birmingham, Birmingham, UK; 6 Birmingham Health Partners Centre for Regulatory Science and Innovation, University of Birmingham, Birmingham, UK

**Keywords:** Patient-reported outcomes (PROs), Clinical trials, Impact

## Abstract

**Background:**

Patient-reported outcomes (PROs) are increasingly collected in clinical trials as they provide unique information on the physical, functional and psychological impact of a treatment from the patient’s perspective. Recent research suggests that PRO trial data have the potential to inform shared decision-making, support pharmaceutical labelling claims and influence healthcare policy and practice. However, there remains limited evidence regarding the actual impact associated with PRO trial data and how to maximise PRO impact to benefit patients and society. Thus, our objective was to qualitatively explore international stakeholders’ perspectives surrounding: **a)** the impact of PRO trial data, **b)** impact measurement metrics, and **c)** barriers and facilitators to effectively maximise the impact of PRO trial data upon patients and society.

**Methods:**

Semi-structured interviews with 24 international stakeholders were conducted between May and October 2018. Data were coded and analysed using reflexive thematic analysis.

**Results:**

International stakeholders emphasised the impact of PRO trial data to benefit patients and society. Influence on policy-impact, including changes to clinical healthcare practice and guidelines, drug approval and promotional labelling claims were common types of PRO impact reported by interviewees. Interviewees suggested impact measurement metrics including: number of pharmaceutical labelling claims and interviews with healthcare practitioners to determine whether PRO data were incorporated in clinical decision-making. Key facilitators to PRO impact highlighted by stakeholders included: standardisation of PRO tools; consideration of health utilities when selecting PRO measures; adequate funding to support PRO research; improved reporting and dissemination of PRO trial data by key opinion leaders and patients; and development of legal enforcement of the collection of PRO data.

**Conclusions:**

Determining the impact of PRO trial data is essential to better allocate funds, minimise research waste and to help maximise the impact of these data for patients and society. However, measuring the impact of PRO trial data through metrics is a challenging task, as current measures do not capture the total impact of PRO research. Broader international multi-stakeholder engagement and collaboration is needed to standardise PRO assessment and maximise the impact of PRO trial data to benefit patients and society.

## Background

Patient-reported outcomes (PROs) are any report of the patients’ perspectives about the impact of disease and treatment on their health status, for example quality of life and symptoms, without the interpretation of a clinician, or anyone else [1, 2]. PRO measures can be classified as disease-specific or generic instruments [3, 4]. The former are customised to specific health conditions, populations or certain functions; such as the Oxford Hip Score (OHS) [4, 5]. Generic instruments focus on general aspects of health-related quality of life, irrespective of the disease or health condition of the patient; such as the 36-Item Short Form Survey (SF-36) [5, 6]. The data from both instruments can be used to demonstrate different types of impact.

Inclusion of PROs in clinical trials can provide unique patient-centred data, which can be used to help clinicians and patients to make more informed treatment decisions, support pharmaceutical labelling claims and influence healthcare policy [7–10].

However, the lack of scientifically rigorous PRO data collection, analysis and reporting is a waste of resources and hinders the maximisation of PRO trial impact [10–13]. Our recent systematic review suggested that PRO trial data have the potential to lead to a range of benefits for patients and society, which can be measured through impact metrics [14]. In addition, stakeholders often focus on narrow and distinct forms of impact [14]. For instance, the PRO data arising from the Cardiac Resynchronisation – Heart Failure (CARE-HF) trial, as measured with the EQ-5D (European Quality of Life Instrument - 5 Dimension) and MLWHF (Minnesota Living with Heart Failure Questionnaire), demonstrated improved symptoms and quality of life [15]. The trial produced 24 publications, of which 4 were PRO-specific. The main trial publication has been cited on 4927 occasions [16] and the findings have led to changes in national and international guidelines and national and international clinical practice [17–19].

To date, however, there has been little research exploring how PRO research impact is realised and measured in practice or the barriers and facilitators to realising this impact. Therefore, the purpose of this study was to qualitatively explore international stakeholders’ perspectives on: **a)** the impact of PRO trial data, **b)** PRO impact metrics to measure such impact, and **c)** barriers and facilitators to effectively maximise the impact of PRO trial data upon patients and society.

## Methods

### Design

A generic qualitative approach was chosen to explore in-depth participants’ perspectives, specifically on the overall impact arising from trial PRO findings, rather than impact associated from a particular PRO measure. Measuring the impact from a single metric does not fully capture the relationships involved in a clinical trial and may exclude some important aspects of the research pathway [20].

This facilitated a rich description of their perspectives while staying close to the data. In addition, this approached was deemed suitable as no theoretical assumptions were made [21, 22]. In order to obtain a broad insight of the participants the expert purposeful sampling method was selected [23]. One-to-one semi-structured interviews were chosen as this qualitative data collection method allows obtaining ‘rich’ data by building a trust relationship with the participants [24]. Finally, the data was analysed using the reflexive thematic analysis method [25–27]. This qualitative study is reported in accordance with the Consolidated criteria for reporting qualitative research (COREQ) [28].

### Ethical considerations

The research followed the University Of Birmingham Code Of Practice for Research and Data Protection and Handling Guidelines [29]. Ethical approval was granted by the University Research Ethics Committee in December 2016 (ERN_16–0806).

### Sampling and recruitment

International stakeholders who spoke English and were willing and able to give informed consent were invited via email to take part in the qualitative interviews. Stakeholders included: policy-makers, representatives from regulatory agencies, funders, journal editors, academic trialists, clinicians and industry trialists. Individuals were eligible for interview if: **a)** they reported experience of using PRO data to inform clinical practice, clinical guidelines and health policy development; to support drug approval, pricing and reimbursement decisions, or to inform clinical decision-making and consent for treatment; or **b)** they reported experience of reviewing the PRO components of clinical trials and/or scientific publications. Initial recruitment approaches were made through personal research networks known to the team (MC/DK/AS) and through the identification of key authors from relevant PRO literature (expert purposive sampling [30]); further participants were identified and recruited through snowball sampling [31].

### Data collection

Semi-structured interviews were conducted by SCR between May and October 2018, either by phone or face to face on University premises [32].

All participants gave either verbal or written informed consent prior to each interview. An interview schedule (Appendix 1) was used to guide the discussion. This was initially informed by our systematic review on PRO trial impact [14]. It was subsequently refined after two pilot interviews with two international stakeholders and consultation with the research team (CM/DK/AS/MC). The aim of the pilot interviews was to identify any flaws or limitations within the interview design. As no major changes to the interview schedule were required, these data were included in the cohort of interviews analysed. Table 1 provides further detail on the topics covered by the interview schedule.

**Table 1:** Summary of interview schedule

Topic area	Summary of subtopics covered
a) The impact of PRO trial results	Exploration of international stakeholders’ perceptions of PRO trial impact, specifically: • Impact of PRO trial data on stakeholder’s practice • Thoughts, opinions and experience of incorporating PRO trial data in practice • Examples of PRO clinical trials that have led to impact • Examples of PRO clinical trials that have not led to impact
b) Impact measurement metrics	Exploration of stakeholders’ perceptions of the most effective ways to identify trials that have led to PRO impact, specifically: • Identify impact measurement metrics • Identify the most effective way to measure PRO trial impact • Thoughts and opinion of developing a framework to measure PRO trial data
c) Perceived barriers and facilitators to effectively maximise the impact of PRO trial data upon patients and society	Exploration of stakeholders’ perceptions of barriers and facilitators to maximise the impact of PRO trial data, specifically: • Thoughts, opinions and experience of facilitators to that maximise the impact of PRO trial data • Thoughts, opinions and experience of barriers to that maximise the impact of PRO trial data

After the piloting exercise, data collection and analysis were conducted iteratively (i.e. themes identified within early interviews and interpreted within transcripts were included in subsequent interviews) until analytic saturation was reached. Saturation is defined as ‘data adequacy’ [33], the point when data collection does not contribute any additional information and the data collected provides comprehensive information to answer the research question [33–35]. For the purpose of this study, saturation was reached when no new themes were interpreted from the data [34, 35]. In this qualitative study, saturation was determined at the stakeholder cohort level through review of the data and discussion within the research team.

### Data analysis

Interviews were digitally recorded and transcribed verbatim by a professional transcription company. Interview data were managed using a qualitative data analysis software package (QSR NVivo 11). Data analysis was informed by the reflexive thematic analysis approach [27]. In order to support the analysis and interpretation of the data, a multidisciplinary team including methodologists, clinical and non-clinical experts was involved. The analysis process started with reading the transcripts several times to increase familiarity with the data. This was followed by deductive and then inductive coding processes.

#### Deductive analysis

Initially, deductive coding was undertaken using the ‘pathways to research impact’ framework [20], in order to identify types of PRO trial impact and impact measurement metrics. The framework provided a comprehensive summary of impact categories, impact subgroups and impact metrics across five types of impact: 1) Primary research related impact; 2) Influence on decision-making; 3) Health and health systems impact; 4) Health-related and societal impact, and 5) Broader economic impact [20] (Appendix Figure 1). The five impact categories of the framework were deductively applied to the data. In instances where it was not possible to categorise data into the existing framework, they were added to a ‘miscellaneous’ coding category. Subsequently, the data coded into each of the impact categories was organised into subgroups.

#### Inductive coding

More detailed codes were described and interpreted inductively within each of the five categories and the ‘miscellaneous’ category. In addition, inductive coding was also used to identify impact metrics, and barriers and facilitators to PRO trial impact, across the whole dataset. After the coding process, and collation of codes, theme generation continued until the definitive overarching themes were developed [25, 27].

Following inductive coding, the transcripts were again read several times to ensure there were no elements of the dataset missing. During the coding stage, a random sample of interviews (10%, *n* = 3) was additionally coded by an independent researcher (CM) in order to enhance credibility of the analysis of the data collected (analyst triangulation) [31]. After the coding process, data were organised and analysed following descriptive accounts by type of impact, impact metrics and barriers and facilitators to impact [36]. Subsequently, a record was included of the meaning of the quotes under each code. These notes helped grouping together descriptive codes that shared common meaning into categories in order to get a broader sense of the data. The final stage was to group relevant categories into themes to represent broader concepts of the data. The themes identified were either rearranged to create a new theme or collapsed to form a single theme [25]. In addition, the themes were revised to ensure they clearly and concisely described the dataset. Quotes that highlighted the nature of each theme vividly were chosen to demonstrate their prevalence. To draw commonalities and differences among stakeholders, descriptive tables were created per theme and subsequently grouped by main topic. The tables included quotes that helped describing the key findings from the dataset. The respective coding, categories and themes decisions were discussed with the research team (DK/AS/MC) to inform the final analysis and interpretation of the data.

## Results

Of 41 stakeholders invited to participate, 24 semi-structured interviews were conducted with a range of international stakeholders. Reasons for declining participated included lack of availability (*n* = 4), preference to maintain a neutral position regarding the topic (*n* = 1), belief they were ineligible (n = 1). In addition, 11 people did not respond to the invitation. Interviews lasted on average 35 min (range 24 to 55 min). Most of the interviews (*n* = 21) were conducted by phone whilst three took place face-to-face on University premises. Interviewees self-identified with a range of stakeholder groups including academic and industrial trialists, journal editors, clinicians, funders and policy-makers/regulators. Six participants identified with more than one group. Participant summary characteristics are presented in Table 2.

**Table 2:** Participants’ characteristics

Stakeholder group	Country	Type of institution	Participant number
**Academic trialists**	USA	University	1
	Australia	University	2
	The Netherlands	University	3
	Canada	University	9
	USA	University	10
**Industry trialists**	USA	Research institute	4
	USA	Research institute	5
	UK	Pharmaceutical company	11
	USA	Pharmaceutical company	16
	USA	Global contract research organisation	17
**Journal editors**	USA	Peer-reviewed medical journal	7
	UK	Peer-reviewed medical journal	15
	USA	Peer-reviewed medical journal	24
**Clinicians**	USA	University	1*
	UK	Government	8*
	Canada	University	9*
	UK	Charity	18*
	USA	Funding institute	19*
	UK	University	23
	UK	University	24*
**Policy-makers and regulators**	USA	Regulatory agency	6
	UK	Regulatory agency	8
	Germany	Reimbursement agency	12
	UK	Regulatory agency	13
	UK	Reimbursement agency	14
**Funders**	UK	Charity	18
	USA	Funding institute	19
	UK	Government	20
	USA	Funding institute	21
	USA	Funding institute	22

*Participant included in two different stakeholder groups

Interpretation of four core themes are presented in this section: 1) types of PRO impact 2) PRO impact metrics and 3) barriers to PRO trial impact, and 4) facilitators to PRO trial impact. To explain the dataset in a meaningful way, an informed approach was adopted. The dataset was presented against these four core themes, which relate back to the five types of impact categories as appropriate throughout.

Results are presented below with quotes labelled as shown in Table 3 followed by participant number. Deviant cases were explored and presented were appropriate.

**Table 3:** Quotes labels

Stakeholder group	Academic trialists	Industry trialists	Journal editors	Clinicians	Policy-makers and regulators	Funders
**Label**	AT	IT	JE	CL	PM-RE	FU

### Types of PRO impact

The following section describes the different types of impact identified by stakeholders in which PRO trial data were purported to have an impact.

#### Primary research related impact

This is an impact associated with the generation of new knowledge, dissemination of results, building of research capacity, delivery of training and development of new leadership, and academic collaborations and networks. This impact is expected to be generated in the short-term, one year or less [20].

Academic and industry trialists, clinicians and funders were the main stakeholder groups that discussed the potential impact of PRO trial findings on ‘research and innovation outcomes’. They believed that publications (including press releases and lay summaries), peer reviewed articles and citation rates have the potential to maximise the impact of PRO trial data outcomes by making the PRO data available to patients, clinicians and decision-makers. Another type of PRO impact mentioned was ‘dissemination and knowledge transfer’, which participants identified as presentation of PRO trial data in conferences by leaders or experts (including patients), mass media, and translation of PRO data to other research areas.

*“The most impactful thing is when a respected expert gets up on the podium and says, “It’s really important that this study showed pain improvements and we should be telling our patients that their pain gets better.” That makes a big difference […]” ****CL1***

See Table 1 in Appendix Table 1 for further quotes on primary research related impact.

#### Influence on policy-making

This type of impact refers to the interaction between policy-makers and academics and available knowledge base, which may result in changes to policy. These impacts are generally considered to arise in the mid-term (1 to 3 years) [20].

Several interviewees highlighted the potential impact of PRO trial data on ‘type and nature of policy-making’, by influencing changes in clinical guidelines to practice and providing information to support drug approval, pharmaceutical labelling claims and promotional labelling claims.

*“[…] to support a drug license, what we would hope in the future is that patient reported outcomes are the patient voices captured in a way, in a robust way, and an objective way that would allow that data to be integrated into the assessment of benefits and risks and then concluding on whether a drug should be given a drug license”****PM-RE13***

For instance, some clinicians stated that PRO trial data had influenced their own practice by informing nuanced conversations with patients and supporting careful selection of treatments and giving them confidence to choose the best healthcare treatment while considering toxicity and side effects.

*“So, for treatments that I discuss with patients, when there are results from trials with information about patient reported outcomes, specifically about symptoms or physical functioning, or overall quality of life I include those in my discussion with patients when they’re making a decision about a treatment.”****CL1***

The impact subgroup ‘type and nature of policy-making’ was mainly discussed by academic trialists, policy-makers and regulators. See Table 2 in Appendix Table 1 for further quotes on influence and policy-making impact.

#### Health and health systems impact

Health and health systems impact encompasses the benefits of health research outputs on `quality of care and service delivering’, ‘evidence-based practice’, ‘improved information and health information management’, ‘cost containment and effectiveness’, ‘resource allocation’, and ‘health workforce’. This type of impact is expected to arise in the long-term, beyond five years [20].

Clinicians and funders were the only stakeholder groups who highlighted the impact of PRO trial data on ‘evidence-based practice’, specifically on the subgroup fulfilling previously unmet needs.

*“Collecting PROMs on a regular basis allowed us to demonstrate that the management of lymphoedema within our organisation was an unmet need, and using that data, we could then use that to influence purchases and commissioners and make that the backbone of a business case which allowed us to provide new services for patients with lymphoedema.”****FU18***

The impact subgroup ‘quality of care and service delivery’ was only discussed among academic and industry trialists, clinicians and funders. These stakeholder groups emphasised the impact of PRO trial data on improved health outcomes. The impact subgroup ‘cost containment and effectiveness’ was predominant among all the stakeholder groups but journal editors. Academic and industry trialists and policy-makers and regulators highlighted the impact of PRO trial data on cost effectiveness.

*“Well, I sub-divide patient reported outcomes into disease specific PROs and generic ones. The generic ones in particular, EQ-5D. All of the trials that we’ve seen that include the EQ-5D have used them to calculate cost effectiveness.”****PM-RE14***

Academic and industry trialists, clinicians, funders and policy-makers and regulators thought that PRO trial data can capture improvements in health-related quality of life that can be used in combination with other clinical outcomes to the contribution of health institutions cost savings. Furthermore, industry trialists, clinicians and funders mentioned that the adoption of a healthcare treatment that improves health-related quality of life could have an impact on the reduction in the number of work loss days, which leads to improved work productivity. The impact sub-category ‘reduction in the number of work loss days’ is encountered within the category ‘healthy workforce’.

*“For irritable bowel syndrome […] patients had less gas and less this and that, but also that led to improvement in work productivity. They went back to work much earlier so that sort of thing certainly has impact in certain segments of the market.”****IT4***

In general, clinicians and funders primarily highlighted the impact of PRO trial data on the ‘health & health systems’ type of impact. Moreover, the impact category ‘resource allocation’ was not discussed by any of the interviewees. See Table 3 in Appendix Table 1 for further quotes on health and health systems impact.

#### Health-related & societal impact

Health-related and societal impact includes the impact subgroups: ‘health literacy’, health knowledge, attitudes and behaviours’ and ‘improved social equity, inclusion or cohesion’. This type of impact is also expected to be generated in the long-term, beyond five years.

Funders and a small number of clinicians highlighted that PRO trial data can influence health literacy, by providing information on how patients are affected by a health condition. They believed that this information can be used to change the general perception of a disease or de-stigmatise it (e.g. cancer and mental health conditions) and help patients ‘live better’ with that condition. Industry trialists and funders considered the impact of PRO trial data on patient advocacy groups, which is encountered within the impact category health knowledge, attitudes and behaviours. These interviewees mentioned that patient advocacy groups can influence drug development by communicating to health authorities patients’ priorities.

*“I think PROs can affect the public image of the disease. I think one of the things we’re all hoping for some day, are treatments for cancer that can help turn what, for many people is a fear of even being with somebody who has cancer into something more positive, that cancer becomes something that we treat like arthritis.”****F21***

Industry trialists and funders were the only stakeholder groups that mentioned the potential impact of PRO trial data on ‘health-related & societal impact’. Furthermore, the impact category ‘improved social equity, inclusion or cohesion’ was not discussed by the interviewees. See Table 4 in Appendix Table 1 for further quotes on health-related and societal impact.

#### Broader economic impacts

This impact category refers to the generation of economic revenue generated from the commercialisation of health research output. This type of impact is also expected to arise in the long term. Industry trialists suggested that PRO trial data can contribute to increasing pharmaceutical companies’ sales and revenue. By using PRO trial data to attract income from intellectual property and increased pharmaceutical sales.

*“Think in the pharmaceutical industry, because we’re selling products, one of the main ways they evaluate whether or not it’s a success is how much it sells, how frequently it’s used and whether or not it becomes part of guidelines, but that’s not the only way to understand the value.”****IT4***

See Table 5 in Appendix Table 1 for further quotes on broader economic impact.

**Fig. 1:**
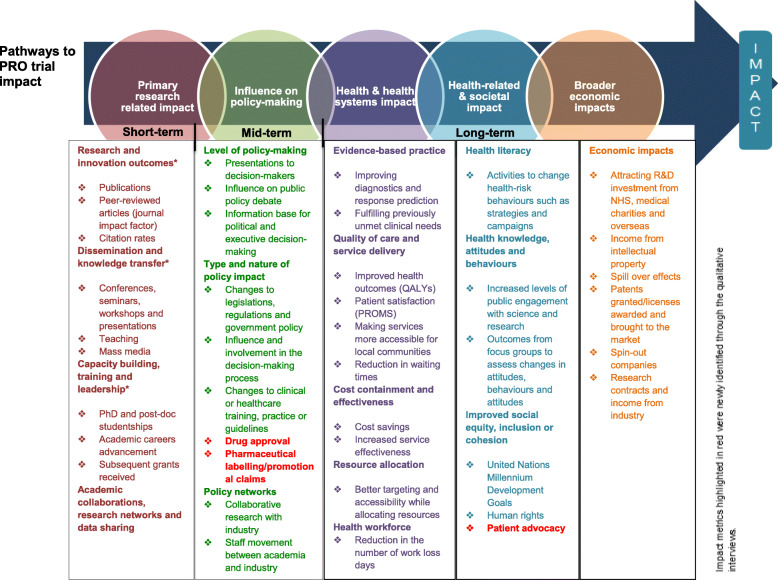
Impact measurement metrics

Figure 1 provides a summary of the different types of PRO impact interpreted within the interview dataset drawn upon the ‘pathways to research impact’ framework [20]. The impact metrics highlighted in different colour represent new areas identified by the dataset.

### Impact measurement metrics

Interviewees proposed different quantitative and qualitative metrics to measure the impact of PRO trial data. These included: the number of citations of PRO publications; journal impact factor; and how often a PRO endpoint was presented in blogs, online communities and social media.

*“How many times the article on the PRO’s has been cited. That’s one measure. The standard metrics that the journals and articles have regarding impact.”****IT16***

Additional impact metrics proposed included: number of clinical trials conducted which included PROs as an endpoint and number of labelling claims. Evaluation of health technology assessment documentation to determine whether PRO data inform drug approval. Surveys and interviews among healthcare practitioners and patients to determine whether PRO data are incorporated into clinical decision-making or used to inform patient shared decision-making.

*“I think there are experimental methods that could be applied, so sampling, […] there’s quantitative survey methods that could be used, but also qualitative methods to be sure to be capturing what its impact is, for instance, how information from a PRO affected thinking and behaviour on the part of the end user. By end user I mean an individual with a condition or a clinician and even to the level of the health system.”****FU21***

In contrast, several stakeholders highlighted that measuring PRO trial data through metrics is a challenging task and it might not accurately represent the real impact of PRO trial data.

### Barriers to PRO impact

Interviewees highlighted a range of perceived barriers that they felt may impair the realisation of impact arising from PRO trial findings, including: 1) poor quality trial design, 2) suboptimal conduct and analysis, 3) poor reporting quality, and 4) dissemination and uptake of PRO results.

#### Poor quality trial design

Poor quality trial design refers to the lack of PRO-specific methodological rigor during the design stage of the clinical trial, which limits the realisation of PRO trial impact in the subsequent stages of the clinical trial. All stakeholder groups, with the exception of industry trialists and funders, mentioned poor quality design as one of the barriers to PRO impact. Interviewees highlighted PRO trial barriers such as lack of detailed PRO protocol and lack of adherence to it.

*“[PROs are] either exploratory endpoints that are either added in inappropriately, the timings are incorrect, the instrument might not be correct for the particular patient population, the analysis hasn’t been thought through, there’s no hypothesis or objectives listed in the study protocol.”****PM-RE13***

Further barriers identified were the inclusion of PROs in the clinical trial as secondary outcome, lack of PRO trial information from phase I and II limiting the design of the PRO component in phase III and; late or not incorporation of PRO experts in the development of the clinical trial protocol.

*"[…] we would get sent the protocol right at the end, right before the trial was going to be sent to ethics or sometimes even after they had received ethical approval. We would make suggestions to improve the protocol with respect to PRO’s and then some of the investigators would be reluctant to make those changes because it meant they would have to do an extensive protocol amendment"****AT2***

See Table 1 in Appendix Table 2 for further quotes on poor quality trial design.

#### Suboptimal conduct and analysis

The way the trial was conducted and the type of analysis that is implemented were mentioned as barriers to maximise the impact of PRO trial data. Suboptimal conduct and analysis was a predominant theme among all the stakeholders; however, it was discussed to a lesser degree by journal editors and clinicians. This theme included barriers related to high rates of missing data, difficulty collecting PRO data among global trials, patient and staff burden and lack of training for clinicians to optimally conduct a PRO clinical trial and analyse PRO trial data and; lack of expert reviewers to assess PRO trial results.

*"Medical journals often lack sufficient experts who can review PRO results because the researchers and clinicians who are journal reviewers are not knowledgeable about PRO[s]."****IT11***

An additional barrier identified surrounded a perceived lack of understanding and interpretation of PRO data by clinicians, patients and patient advocates.

*“PRO experts, sometimes assume that the clinicians will understand tables and figures and the interpretation of the clinical trial and I think we know from experience that clinicians don’t always get the message.”****IT17***

See Table 2 in Appendix Table 2 for further quotes on suboptimal conduct and analysis.

#### Poor reporting quality

This theme was primarily highlighted by academic trialists. Barriers emphasised by interviewees included: lack of discussion of PRO outcomes; inclusion or detailed information within the main clinical trial publication; PRO data explanation in view of other clinical endpoints and; publication of PRO trial data many years after publishing the main trial manuscript or its lack of publication.

*“I came across a few trials where the PRO results hadn’t been published […] I saw that certain trials had PRO of secondary endpoint but then when I found the publication that related to it, that was just completely missing and sometimes they would say that the PRO results would be published later but it had been several years down the track.”****AT2***

Further barriers to PRO impact interpreted included publication of clinical trials manuscripts including PRO data in a technically correct language, but difficult to understand for patients, advocacy groups and patients and; restricted access to PRO publications (paywall restrictions). See Table 3 in Appendix Table 2 for further quotes on poor reporting quality.

#### Dissemination and uptake of PRO results

This included PRO-specific issues faced upstream that limited the propagation and adoption of the findings into clinical practice. This theme was common among journal editors, clinicians and policy-makers and regulators, whereas academic trialists commented on this theme to a lesser extent.

Barriers encompassed lack of awareness of PRO data importance between clinicians, researchers, journal editors and sponsors and; prioritisation of clinical outcomes over PRO trial data by researchers and funders.

*“The main reason is that the high impact journals want survival data and if they’ve got a survival advantage they don’t bother with the quality of life data. […] There’s a study of a drug which has a two-month survival advantage, worse toxicity, quality of life data collected but not published. It’s outrageous.”****FU19***

Additional barriers discussed were lack of engagement between academic researchers and research companies with patients to understand patient priorities, collaboration between PRO researchers within same health research areas and law or regulation in the UK to enforce the collection of HRQL in clinical trials.

*"So we are law enforcers if you like. Now, that doesn’t really incorporate PRO’s, there is no specific law if you like, that they’re going to break if they don’t include a PRO or include it in the wrong context or what have you. So it’s almost, I suppose, supplementary information. It’s not regulated in any way in terms of black and white text."****PM-RE8***

Other barriers that reportedly might hinder the maximisation of PRO trial data were the limitation of PROs not automatically becoming health utilities, the different perspective surrounding the inclusion of PROs in clinical trials between the EMA (European Medicines Agency) and FDA (Food and Drug Administration) and the difficulty getting funding for PRO research.

*“One of the major funders of research in this country is very unlikely to fund research that has a PROM as a primary outcome. They’ve made that a strategic intent, so the playing field is already biased against PROM’s based research.”****FU18.***

See Table 4 in Appendix Table 2 for further quotes on dissemination and uptake of PRO results.

### Facilitators to PRO impact

Interviewees highlighted a range of perceived facilitators that they felt may enhance the realisation of PRO trial findings including: 1) improved PRO trial design, 2) optimal conduct and analysis, 3) improved reporting and 4) dissemination and uptake of PRO results.

#### Improved PRO trial design

This theme was primarily discussed by policy-makers and regulators. It was not discussed among journal editors and clinicians. Improved PRO trial design facilitators discussed by interviewees included: the production of a clear and detailed PRO protocol; endorsement of the PRO data as a key endpoint in clinical trials; and early incorporation of a PRO expert in the trial team.

*"There should be a PRO expert on the clinical trial team and at the earliest possibility; if you start thinking about your PROs at the reporting stage it is far too late. You need to be thinking much earlier on."****AT3***

Participants also discussed adherence to PRO guidelines, inclusion of patients and clinicians in the trial design stage, regular meetings with regulatory agencies during the planning period and the end of the trial and; the development of PRO measures while considering health utilities for HTA use.

*"The patient reported outcomes world could think of utilities at the same time as developing their PRO’s. So any patient reported outcome that has got a utility mapping attached to it is very useful."****PM-RE14***

See Table 1 in Appendix Table 3 for further quotes on improved PRO trial design.

#### Optimal conduct and analysis

Facilitators to achieve optimal conduct and analysis were highlighted by policy-makers and industry trialists, whereas journal editors and clinicians did not contribute to this theme. Facilitators discussed were high completion rates of PRO trial, training sites on the administration, make PRO data more readily understandable and; explanation of PROs and standardisation of PRO tools among therapeutic areas to improve analysis.

*"Having standardised tools across trials helps us understand the trial results and be able to compare things more easily. [...] Certainly in the US with qualification process for PROs, there’s a hope that each of us will not go out and create one off our own tool, instead have some standardisation."****IT5***

See Table 2 in Appendix Table 3 for further quotes on optimal conduct and analysis.

#### Improved reporting

Improved reporting was predominant among academic trialists and journal editors; however, this theme was not discussed by industry trialists, policy-makers, and regulators. Facilitators encompassed in this theme were open access publications and PRO trial data reported in the main publication and in a high impact journal.

*"The studies which have been impactful have been ones where the quality of life data and the survival data has been published together in a high impact journal"****FU19***

Further facilitators comprised simple English summary of the trial results for use of patients, availability of more journals to publish PRO trial data and make PRO instruments available through publications. See Table 3 in Appendix Table 3 for further quotes on improved reporting.

#### Dissemination and uptake of PRO results

Finally, dissemination and uptake of PRO results was emphasised by all the stakeholders but to a lesser extent by policy-makers and regulators. This theme highlighted facilitators such as adequate funding and the important of funders clearly stating their position around PROs and their expectations of the funded PRO research.

*“I think funders have a role because they can stipulate, for example, that the work they fund must have some sort of an implementation plan so that work isn’t just completed and then perhaps published in a journal and then never heard from again. Having emphasis on ensuring that there is some pull through into use and impact as a direct requirement of funding would go a long way as well to help the problems.”****FU21***

In addition, it was suggested that funders should require an implementation plan in terms of usage and impact of the PRO clinical trial as a direct requirement of funding. Facilitators suggested that might enable the dissemination and uptake of PRO results included: provide PRO training courses for clinicians and drug developers and communicate PRO research widely through the involvement of key opinion leaders, specifically at healthcare conferences.

*"To allow organisations like the NCRI, ASCO, ESTRO, the organisations that host large healthcare provider conferences to make PROM’s based research a future of their sessions and their main talks and also to improve quality of science communications so that we have skilled science communicators disseminating these results."****FU18***

Further facilitators highlighted included empowerment of patients through their involvement in discussions and dissemination of PRO trial results and; endorsement of PRO trial studies by key societies to disseminate results and influence healthcare policy. See Table 4 in Appendix Table 3 for further quotes on dissemination and uptake of PRO results.

## Discussion

For the first time, this study provides international stakeholder perspectives on the types of impact associated with PRO trial results, impact measurement metrics, and barriers and facilitators to effectively maximise the impact of PRO trial data upon patients and society.

Stakeholders identified a number of ways in which PRO data from clinical trials can potentially inform/influence primary research, policy-making, health and health systems, health-related and societal impact and broader economic impacts. Although every interviewee was asked similar questions, not all of them discussed each type of impact. It was interpreted from the data that stakeholders appeared to focus on the impact categories that were most relevant to them and did not focus on broader aspects of PRO impact, even when prompted. The dataset provided rich narratives when the interviewee had experience of a particular type of PRO impact. For instance, academics primarily focused on ‘primary research related impact’. Arguably this stakeholder group might be more focused on producing research outcomes and their dissemination, rather than the broader benefits these outcomes may have on patients and society. Nonetheless, PRO stakeholders agreed on the benefit of including PROs in clinical trials and did consider a range of impacts.

The majority of the stakeholders suggested that measuring the impact of PRO trial research can benefit academic researchers, trialists, policy-makers, regulatory authorities, funding bodies, pharmaceutical companies, payers and patients. Measuring the impact of PRO trial findings may help stakeholders understand the importance and value of PRO trial data, broaden their perspectives regarding PRO applicability, and identify the different benefits to society through improved health outcomes and use of resources [37]. For instance, these data would provide a knowledge base to policy-makers, regulators and funders to justify drug approval and inform funding allocation decisions through demonstrating the potential benefits on patients and society [20, 38]. Moreover, journal editors and academics might be more likely to acknowledge the importance of PRO data and ensure timely, transparent publication of PRO trial data in high impact journals. Considering the impact of PRO trial impact, it has the potential to influence the study design and determine the possible benefits of conducting a particular study. Table 4 provides an example of PRO clinical trial that led to impact.

**Table 4:** Impact of PRO trial data: a practical example

Tocilizumab, a new treatment for rheumatoid arthritis (RA) in adults and juvenile idiopathic arthritis (JIA) in children showed significant improvements.	
Patients improved 30% or more on at least three of the six variables in the American College of Rheumatology (ACR) core set for JIA, with no more than one variable worsening by more than 30%. Furthermore, patients showed improved symptoms such as absence of fever and rash, as measured with the Disability Index of the Childhood Health Assessment Questionnaire (CHAQ-DI) and; improved laboratory abnormalities (anemia, thrombocytosis, and hyperferritinemia) – primary outcome [39].	
**Drug approval:** Tocilizumab was approved in 2009 by the EMA (European Medicines Agency) [40] and by the FDA in January 2010 [41] for use in RA based on clinical and PRO findings.	
**Health improvements:** Tocilizumab has been supported for prescription by NICE in the UK for patients with severe RA. There is evidence that tocilizumab halts joint damage, improves function and increases quality of life [39]. Significantly more patients treated with tocilizumab showed improvements of ≥0.3 units in the HAQ-DI score compared to patients treated with placebo [39].	
**Informed cost-effectiveness:** The National Institute for Health and Clinical Excellence (NICE) recommended the use of tocilizumab for systemic onset JIA, the most severe form of JIA, in 2011. The manufacturer’s submission mapped the CHAQ-DI scores to utilities, using a mapping formula derived in adults with rheumatoid arthritis that mapped Health Assessment Questionnaire [HAQ] results onto EQ-5D utilities to determine the effectiveness of the intervention [42].	
**Reduction in the number of work loss days:** according to a case study presented by the National Rheumatoid Arthritis Society, patients under tocilizumab are able to return to work as the drug halts joint damage and improves function [43].	
**Income from the intellectual property:**Roche, manufacturer of the drug, reported 496 m CHF (£335 m) in sales of the drug in just the first half of 2013 (up 33% on the previous year, due to increasing demand) [44].	

Furthermore, stakeholders proposed several qualitative and quantitative metrics to measure the impact of PRO research. Quantitative metrics included number of publications, citations including PROs as an endpoint and number of regulatory approvals including PROs and; surveys among stakeholders and patients to determine how PRO data are being used. Qualitative metrics comprised interviews among people involved in the drug approval process to determine whether PRO trial data inform drug approval appraisal. Certain metrics may be more important to particular stakeholders but should be considered for academic and industry trials collecting PROs.

However, most interviewees highlighted that measuring the impact of PRO trial research is a challenging task as it cannot be captured systematically. Single cross-sectional metrics tend not to represent the overall impact PRO trial data can have, since impact arises at different points in time [20, 45]. In addition, impact is defined by each stakeholder group in a different way. For instance, academics considered impact in terms of number of publications and journal impact factor; policy-makers and regulators in terms of changes to healthcare policy and number of drug approvals. The most appropriate way to measure PRO trial data will depend on each stakeholders’ needs. The ‘pathways to research impact framework’ [20] proposes different quantitative and narrative metrics to measure such impact; however, research teams may wish to use a multidimensional approach that may present a more comprehensive method of measuring impact. Nonetheless, further work should be done to determine the effectiveness of the impact metrics identified.

Several methodological PRO-specific trial barriers were identified including poor quality trial design, suboptimal trial conduct and analysis and poor reporting quality. Interestingly, funders did not raise poor quality trial design as an issue in their interviews but arguably should be concerned with the quality of the data collected, as it is considered a crucial barrier to the realisation of PRO trial impact downstream. It is important that trialists consider the rationale for PRO assessment at the start of the study and select appropriate measures to help realise the impact that they wish to achieve. For example, use of health utility measures to inform CE analyses and disease specific measures to inform clinical practice.

Facilitators to maximise the impact of PRO trial data were discussed among stakeholders. Main facilitators highlighted were: mandatory inclusion of PRO data in funded trials and publications where appropriate; and the requirement to provide an implementation plan detailing the proposed use and impact of PRO clinical trial data as a direct requirement of funding. Additional facilitators included the importance of communicating PRO research widely, specifically at healthcare conferences hosted by organisations such as NCRI (The National Research Cancer Institute), ASCO (American Society of Clinical Oncology), and ESTRO (European Society for Radiotherapy & Oncology). It also felt important to empower patients by including them in the dissemination of PRO results at these healthcare conferences. Furthermore, the development of a UK law to enforce the collection of PRO data among clinical trials is considered as essential.

Currently, PRO stakeholders are making concerted efforts to improve the collection of PRO data in oncology and cardiology areas. For instance, the European Society of Cardiology (ESC) has led initiatives to increase the prominence of PROs in cardiovascular research, which can be translated in benefits for patients, clinicians, payers and policy-makers [46]. The Food and Drug Administration (FDA) is currently developing patient-focused drug development (PFDD) guidance to address how stakeholders can collect and include PROs from patients and caregivers in the development and regulation of medical products [47]. In 2016, the EMA published Appendix 2 to the guideline on the evaluation of anticancer medicinal products in man. This provides a general overview of the use of PRO endpoints in oncology studies and the value of this information from the regulatory perspective [48].

Additional initiatives include PROTEUS Consortium (Patient-Reported Outcomes Tools: Engaging Users & Stakeholders) [49], which aims to promote the uptake and use of tools to support high quality PRO trial data including tools such as: SPIRIT (Standard Protocol Items: Recommendations for Interventional Trials) PRO-Extension [12]; ISOQOL (International Society for Quality of Life Research) Minimum Standards for PRO Measures in patient-centered outcomes and comparative effectiveness research [50]; SISAQOL (Setting International Standards in Analysing Patient-Reported Outcomes and Quality of Life Endpoints Data) [51]; CONSORT (Consolidated Standards of Reporting Trials) PRO-Extension [52] Stakeholder-Driven, Evidence-Based Standards for Presenting PROs in Clinical Practice [53]; and Clinician’s Checklist for Reading and Using an Article About PROs However, greater work needs to be done to capture PRO data in a rigorous efficient way across disciplines. Furthermore, key societies like Macmillan Cancer Support, ASCO and the NCRI are working on the endorsement of the dissemination of PRO trial studies, which might help to have a wider reach for spreading PRO trial results and consequently a further impact [54, 55].

### Strengths and limitations

One of the key strengths of this study was the inclusion of 24 internationally recognised PRO experts. We consider the interviews captured all the core concepts around the impact of PRO trial data, which are presented above in four different themes. SCR, the interviewer, did not have a relationship with the participants; however, the wider team (MC/DK/AS) had previous collaborative links with some of the participants. To reduce the potential misinterpretation of data, the multidisciplinary team provided support for the analysis and interpretation of the data.

A further limitation was that since participants were recruited from a pool of stakeholders known to the research team, this might have limited the range and experience of stakeholders being interviewed. Seventeen invitees declined to participate in the research, which could have led to the exclusion of relevant individuals with a different perspective. Whilst we did not ask for a reason why they declined participation, it is possible that they may have been less aware of the potential benefits of PRO trial data and/or the importance of sharing their views irrespective if they did or did not see the benefits of these data.. Nonetheless, we attempted to interview as wide a range of stakeholders as possible using purposive and snowball recruitment methods.

Saturation within each stakeholder group would have been ideal but this was not possible. This would have allowed stronger conclusions to be drawn around the similarities and differences between each stakeholder group. Where there was appropriate evidence, similarities and differences were highlighted. Qualitative experts were involved to ensure congruence and structure at each stage of the research.

Finally, the findings drawn from this study may be transferable to other researchers working on PRO clinical trials, who have knowledge of the area. This reflects the need to have a PRO expert as part of the clinical trial team and; make the research accessible and applicable across a broad spectrum of international stakeholders.

## Conclusion

In this study, we have presented the perspectives of international PRO stakeholders on the impact of PRO trial data, impact measurement metrics, and barriers and facilitators to effectively maximise the impact of PRO trial data upon patients and society. Interviewees highlighted a range of potential impacts associated with PRO trial findings, most notably the influence on policy-making. However, there is a need to find more comprehensive ways of measuring PRO impact. There a number of barriers that needs to be overcome to facilitate PRO impact. Stakeholders need to come together to address these challenges in order to optimise the uptake of PRO trial findings in practice and maximise the benefit to patients and society.

## Supplementary information

**Additional file 1: Appendix 1.**

**Additional file 2: Appendix 2.**

**Additional file 3 Appendix 3**

**Additional file 4: Appendix 4.**

**Additional file 5: Appendix 5.**

## Abbreviations

**ASCO**: American Society of Clinical Oncology
**CARE-HF**: CArdiac Resynchronisation – Heart Failure
**CHAQ-DI**: Disability Index of the Childhood Health Assessment Questionnaire
**CONSORT PRO-Extension**: Consolidated Standards of Reporting Trials PRO-Extension
**COREQ**: Consolidated criteria for reporting qualitative research
**EQ-5D**: European Quality of Life Instrument - 5 Dimensions
**EMA**: European Medicines Agency
**ESC**: European Society of Cardiology
**ESTRO**: European Society for Radiotherapy & Oncology
**FDA**: Food and Drug Administration
**ISOQOL**: International Society for Quality of Life Research
**JIA**: Juvenile Idiopathic Arthritis
**MLWHF**: Minnesota Living with Heart Failure Questionnaire
**MFSAF**: Myelofibrosis Symptom Assessment Form
**NCRI**: The National Research Cancer Institute
**NICE**: National Institute for Health and Clinical Excellence
**PFDD**: Patient-focused drug development
**PROs**: patient reported outcomes
**PROTEUS**: Patient-Reported Outcomes Tools: Engaging Users & Stakeholders
**RA**: Rheumatoid arthritis.
**SISAQOL**: Setting International Standards in Analysing Patient-Reported Outcomes and Quality of Life Endpoints Data
**SPIRIT PRO-Extension**: Standard Protocol Items: Recommendations for Interventional Trials PRO-Extension
