# Supplementary material for: The impact of patient-reported outcome data from clinical trials: perspectives from international stakeholders
Source: J Patient Rep Outcomes. 2020 Jul 2;4:51. doi: 10.1186/s41687-020-00219-4 (PMC7332593; doi:10.1186/s41687-020-00219-4)
Supplement: Supplementary file 1 — Additional file 1: Appendix 1. [file 41687_2020_219_MOESM1_ESM.docx]

**Appendix 1**

**Topic Guide**

**Consent: We are conducting the research** how do patient-reported outcomes (PRO) trial results impact on future patient care and healthcare decisions.

If you agree, I would like to ask you some questions about this topic. The interview should take up to **45 minutes**. Your responses are **confidential and anonymized**. You are able to **withdraw** at any moment during and after the interview, up to 10 working days, without giving a reason.

- Have you received and read the participant’s information sheet?
- Do you have any questions about the study or the interview process?
- Are you willing to proceed with the interview and allow me to use your anonymized data to inform the study and any future publications?
- Would you like have access to any publications that will arise from this research project?
  - **If YES to above**
- Are you happy for me to contact you using the email address you have provided?

**Aim of the study:** i) to explore in depth the impact of patient-reported outcome (PRO) trial results on clinical practice, clinical guidelines and health policy development, drug approval, pricing and reimbursement decisions, clinical decision-making and consent for treatment. ii) To explore perceived barriers and facilitators of effective dissemination and impact on healthcare decisions and patient care. iii) Ultimately the results of this qualitative study will help inform the development of a PRO impact metrics framework.

***By impact****,* ***we mean a positive change or benefit on the economy, society, health, policy and academia.***

**May I proceed with the first question?**

**Background:**

1. Could you tell me about your background and area of research expertise?
2. Do you think PROs trial findings have influenced your own practice?

***If yes****, can you describe any examples?*

- 1. *How did PRO trial findings influence your practice?*
  2. *Why do you think it was important to incorporate PRO trial findings in your practice?*
  3. *Who benefited from the incorporation of PROs?*
  4. *What were the benefits of incorporating PRO trial findings in your practice?*
  5. *When was the trial conducted?*

***If not****, why do you think PRO trial findings do not influence your practice?*

1. Can you think of a specific PRO trial that has led to impact? This could be a trial you have been directly involved or you are aware of.
   1. *Were you involved in this trial?*
   2. *When was the trial conducted?*
   3. *What was the clinical area?*
   4. *Tell me about the impact, why do you think this PRO trial led to impact? (pricing decisions, clinical practice, health policy, clinical guidelines or reimbursement decisions)*
   5. *How did PROs were incorporated?*
   6. *How did PROs facilitate the impact?*
2. In your experience, what is the most effective way to identify an impactful PRO clinical trial that could influence practice? *If necessary*, *use as further explanation for the participant. Presentation of trial results at conferences, publications or clinical practice guidelines.*
   1. *Why do you think this is the most effective way?*
   2. *What are the advantages of this method?*
   3. *What are the disadvantages of this method?*
3. Can you think of any other effective ways to identify impactful PRO clinical trials?

**Barriers and facilitators:**

We are interested in identifying what barriers and facilitators influence the use of PRO trial findings in healthcare decisions and patient care.

1. Thinking about the same impactful PRO trial, what were the **main key things or** **facilitators** that helped its dissemination and uptake in practice?

*If necessary, rephrase. Assuming we have a well conducted study. How can we maximise the benefit of PRO data and inform patient care and healthcare practice?*

1. Can you think of a clinical trial collecting PROs that **has not** led to PRO research impact? This can be a clinical trial you have been involved with or you are aware of.

***If yes,*** *can you describe it?*

- 1. *Why did not it lead to impact?*
  2. *What were the consequences of conducting a PRO trial that does not lead to impact?*
  3. *What were the main limitations of those PRO trial findings?*

1. In your experience, what are the potential **barriers** that prevented the impact of the previous PRO trial results? *If necessary, use as further explanation for the participant. These can be related to trial design, conduct and analysis, reporting, uptake in practice and other factors.*
2. Do you have any suggestions on how best to address barriers to realising PRO impact, or any ideas on how to proactively facilitate such impact? *If necessary, use as further explanation for the participant. Clinicians training, use of SPIRIT and SPIRIT-PRO, CONSORT and CONSORT-PRO*

**Impact metrics:**

We are interested in identifying ways that determine the impact of PRO trial findings have on healthcare decisions and patient care.

1. How could we measure the impact of PRO trial findings on healthcare decisions and patient care in future?

*If necessary, example of metric: FDA – number of labelling claims approved*

1. What ways to measure impact would be the most representative?
2. Do you think it would be useful to have a framework compiling the different measures mentioned?
   1. *Why do you think it would be useful?*
   2. *What are the benefits of having this framework?*
   3. *Who do you think will benefit from this framework?*
   4. *How would it benefit you or other stakeholders involved in PRO trial research?*
   5. *When would it be useful for PRO trial research to use this framework? (Before or after conducting a trial)*
3. Are you aware that this sort of information is being captured?
   1. *If yes, can you give me some examples?*

**Conclusion:**

- I have no more questions, but I would like to give you the opportunity to add anything else we have not discussed.
- Thank you for taking part in this interview. If you have any further questions or comments, please don’t hesitate to contact me.
