# Supplementary material for: The impact of patient-reported outcome data from clinical trials: perspectives from international stakeholders
Source: J Patient Rep Outcomes. 2020 Jul 2;4:51. doi: 10.1186/s41687-020-00219-4 (PMC7332593; doi:10.1186/s41687-020-00219-4)
Supplement: Supplementary file 2 — Additional file 2: Appendix 2. [file 41687_2020_219_MOESM2_ESM.docx]

**
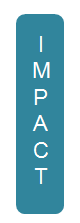
**

Key: [Bold, [impact categories]; Diamond, [impact subgroups]; *top three metrics]

**Level of policy-making**

- Presentations to decision-makers
- Influence on public policy debate
- Information base for political and executive decision-making

**Type and nature of policy impact**

- Changes to legislations, regulations and government policy
- Influence and involvement in de decision-making process
- Changes to clinical or healthcare training, practice or guidelines

**Policy networks**

- Collaborative research with industry
- Staff movement between academia and industry

**Appendix 2**

**Pathways to Impact**

**Economic impacts**

- Attracting R&D investment from NHS, medical charities and overseas
- Income from intellectual property
- Spill over effects
- Patents granted/licenses awarded and brought to the market
- Spin-out companies
- Research contracts and income from industry

**Health literacy**

- Activities to change health-risk behaviours such as strategies and campaigns

**Health knowledge, attitudes, behaviours and outcomes**

- Increased levels of public engagement with science and research
- Outcomes from focus groups to assess changes in attitudes, behaviours and attitudes

**Improved social equity, inclusion or cohesion**

- Assessment of social welfare such as improvements in maternal health and child mortality

**Evidence-based practice**

- Improving diagnostics and response prediction
- Fulfilling previously unmet clinical needs

**Quality of care and service delivering**

- Improved health outcomes (QALYs)
- Patient satisfaction
- Making services more accessible and for local communities
- Reduction in waiting times

**Cost containment and effectiveness**

- Cost savings
- Increased service effectiveness

**Resource allocation**

- Better targeting and accessibility while allocating resources

**Health workforce**

- Reduction in the number of work loss days

**Research and innovation outcomes***

- Publications
- Peer-reviewed articles (journal impact factor)
- Citation rates

**Dissemination and knowledge transfer***

- Conferences, seminars, workshops and presentations
- Teaching
- Mass media

**Capacity building, training and leadership***

- PhD and post-doc studentships
- Academic careers advancement
- Subsequent grants received

**Academic collaborations, research networks and data sharing**

*** Top three impact metrics**

**Long-term**

**Mid-term**

**Short-term**
