# Supplementary material for: The impact of patient-reported outcome data from clinical trials: perspectives from international stakeholders
Source: J Patient Rep Outcomes. 2020 Jul 2;4:51. doi: 10.1186/s41687-020-00219-4 (PMC7332593; doi:10.1186/s41687-020-00219-4)
Supplement: Supplementary file 3 — Additional file 3 Appendix 3 [file 41687_2020_219_MOESM3_ESM.docx]

| **Types of impact** | | | **Discussed by** | | | | | | | **Illustrative quotes** | |
| --- | --- | --- | --- | --- | --- | --- | --- | --- | --- | --- | --- |
|  |  |  | Academic trialists | Industry trialists | Journal editors | Clinicians | Funders | Policy-makers and regulators |  | |  |
| **Primary research related impact** | Research and innovation outcomes | Publications | *✓* | *✓* | *✓* | *✓* |  | *✓* | *“[…] publications are there in the literature to be read. So, I think those are important, and in publication in high ranking journals like New England or Lancet are obviously more important.”* ***CL9*** | |  |
|  | Dissemination and knowledge transfer | Conferences, seminars, workshops and presentations |  | *✓* |  | *✓* | *✓* |  | *“It needs someone like NCRI or NIHR or Evolve or someone just to put a call out. If you’ve had a study that’s improved something via PRO measures, if PROM’s have made an impact tell us what the study was, tell us what it is, and by the way we’re looking to do a showcase for you to talk about it at this or that conference on this or that date.”****JE15*** | |  |
|  |  | Teaching |  |  |  |  |  |  |  | |  |
|  |  | Mass media | *✓* |  | *✓* | *✓* |  |  | *“It could also be TV, radio; newspaper advertisements and other form of media are also ways to spread the knowledge about the PRO’s and to advance the matter so that different people with little understanding of statistics and epidemiology can understand.”* ***IT16*** | |  |
|  |  | Translation of PRO data to other research areas | *✓* |  |  |  | *✓* |  | *“It can also be that it will be translated, or that it will be applied in another field or it influences further research.”* ***AT3*** | |  |
|  | Capacity building, training and leadership |  |  |  |  |  |  |  |  | |  |
|  | Academic collaborations, research networks and data sharing |  |  |  |  |  |  |  |  | |  |

**Appendix 3**

**Table 1 - Primary research related impact quotes**

| **Table 2 - Influence on policy-making impact quotes** | | | | | | | | | |
| --- | --- | --- | --- | --- | --- | --- | --- | --- | --- |
| **Types of impact** | | | **Discussed by** | | | | | | **Illustrative quotes** |
|  |  |  | Academic trialists | Industry trialists | Journal editors | Clinicians | Funders | Policy-makers and regulators |  |
| **Influence on policy-making** | Level of policy-making |  |  |  |  |  |  |  |  |
|  | Type and nature of policy impact | Changes to legislations, regulations and government policy |  |  |  |  |  |  |  |
|  |  | Influence and involvement in the decision-making process |  |  |  |  |  |  |  |
|  |  | Changes to clinical or healthcare *practice* | *✓* | *✓* | *✓* | *✓* | *✓* | *✓* | *“So there was a study where, if you like, the cancer control outcomes were the same but the PROM’s were improved with radiotherapy. That’s a trial that’s influenced my practice and makes me feel very confident to offer radiotherapy in preference to surgery for those patients based on a PROM outcome.”* ***CL18*** |
|  |  | Changes to clinical or healthcare *guidelines* | *✓* |  |  | *✓* |  | *✓* | *“When Mitoxantrone was approved in 1996, I think around that time anyway, I mean yes, guidelines for treatment of metastatic prostate cancer changed to include Mitoxantrone as a treatment, a recommended treatment for patients who develop hormone resistant prostate cancer.”* ***CL9*** |
|  |  | Drug approval* | *✓* |  | *✓* |  |  | *✓* | *“So when we’re weighing up the efficacy data and the safety data, to support a drug license, what we would hope in the future is that patient reported outcomes are the patient voices captured in a way, in a robust way, and an objective way that would allow that data to be integrated into the assessment of benefits and risks and then concluding on whether a drug should be given a drug license”* ***PM-RE13*** |
|  |  | Pharmaceutical labelling claims* | *✓* |  |  | *✓* |  | *✓* | *“In the case of Abiratone Acetate, it was such an important endpoint that it’s included in the drug label for the Food and Drug Administration.”* ***CL1*** |
|  |  | Promotional labelling claims* |  | *✓* |  |  |  | *✓* | *“Of the labelling and so that’s going to be different by country because in the US we have direct consumer advertising, so PRO messaging can go right to the patient, and then patient might go to the doctor and say ‘I saw this commercial and it says I would have improved physical function if I take this migraine medication’.”* ***IT5*** |
|  | Policy networks |  |  |  |  |  |  |  |  |

| **Table 3 - Health and health systems impact quotes** | | | | | | | | | |
| --- | --- | --- | --- | --- | --- | --- | --- | --- | --- |
| **Types of impact** | | | **Discussed by** | | | | | | **Illustrative quotes** |
|  |  |  | Academic trialists | Industry trialists | Journal editors | Clinicians | Funders | Policy-makers and regulators |  |
| **Health & health systems impact** | Evidence-based practice | Improving diagnostics and response prediction |  |  |  |  |  |  |  |
|  |  | Fulfilling previously unmet needs |  |  |  | *✓* | *✓* |  | *“Collecting PROM’s on a regular basis allowed us to demonstrate that the management of lymphoedema within our organisation was an unmet need, and using that data, we could then use that to influence purchases and commissioners and make that the backbone of a business case which allowed us to provide new services for patients with lymphoedema.”* ***FU18*** |
|  | Quality of care and service delivery | Improved health outcomes | *✓* | *✓* |  | *✓* | *✓* |  | *“We looked at enhanced recovery in people undergoing anterior resections of the rectum using laparoscopic surgery and enhanced recovery methodologies […] it was the fact that the patient reported outcomes in the subsequent weeks and months post operatively demonstrated a much quicker return to a high quality of life as opposed to open surgery.”****IT4*** |
|  |  | Patient satisfaction |  |  |  |  | *✓* |  | *“How satisfied [patients] are but also more detailed appropriate patient related outcomes. […] We have to find qualitative systematic ways for doing that because what you want to achieve is the same level of satisfaction but the outcome for individuals could be completely different to maintain that level of satisfaction.”* ***FU20*** |
|  |  | Making services more accessible for local communities |  |  |  |  |  |  |  |
|  |  | Reduction in waiting times |  |  |  |  |  |  |  |

| **Types of impact** | | | **Discussed by** | | | | | | **Illustrative quotes** |
| --- | --- | --- | --- | --- | --- | --- | --- | --- | --- |
|  |  |  | Academic trialists | Industry trialists | Journal editors | Clinicians | Funders | Policy-makers and regulators |  |
| **Health-related & societal impact** | Health literacy | Activities to change health-risk behaviours | *✓* |  |  |  | *✓* |  | *“A field can be changed in terms of the way in which the impact of the condition is on an individual or on a population or on a health system is conceptualised and patient reported measures can have a huge role to play in that […] I think there’s good examples in urinary incontinence therapeutic area that some of the measures that have been developed have changed the way in which the disorder is conceptualised.”* ***F21*** |
|  | Health knowledge, attitudes and behaviours |  |  |  |  |  |  |  |  |
|  | Improved social equity, inclusion or cohesion | Patient advocacy groups* |  | *✓* |  |  | *✓* |  | *“the efforts being made to translate clinical trial outcomes by patient advocacy groups in terms that a patient can understand. So when they go to their clinician they ask for this information or if they know something about the outcomes for the treatment.”* ***IT17*** |

**Table 4 - Health-related and societal impact quotes**

**Table 5 - Broader economic impact quotes**

| **Types of impact** | | | **Discussed by** | | | | | | **Illustrative quotes** |
| --- | --- | --- | --- | --- | --- | --- | --- | --- | --- |
|  |  |  | Academic trialists | Industry trialists | Journal editors | Clinicians | Funders | Policy-makers and regulators |  |
| **Broader economic impact** | Economic impacts | Income from intellectual property |  | *✓* |  |  |  |  | *“Think in the pharmaceutical industry, because we’re selling products, one of the main ways they evaluate whether or not it’s a success is how much it sells, how frequently it’s used and whether or not it becomes part of guidelines, but that’s not the only way to understand the value.”* ***IT11*** |
