# Supplementary material for: The impact of patient-reported outcome data from clinical trials: perspectives from international stakeholders
Source: J Patient Rep Outcomes. 2020 Jul 2;4:51. doi: 10.1186/s41687-020-00219-4 (PMC7332593; doi:10.1186/s41687-020-00219-4)
Supplement: Supplementary file 4 — Additional file 4: Appendix 4. [file 41687_2020_219_MOESM4_ESM.docx]

**Appendix 4**

**Table 1 - Poor quality trial design quotes**

| **Trial stage** | **Barriers** | **Discussed by** | | | | | | **Illustrative quotes** |
| --- | --- | --- | --- | --- | --- | --- | --- | --- |
|  |  | Academic trialists | Industry trialists | Journal editors | Clinicians | Funders | Policy-makers and regulators |  |
| **Design** | Lack of detailed PRO protocol |  |  | *✓* |  |  | *✓* | *“[PROs are] either exploratory endpoints that are either added in inappropriately, the timings are incorrect, the instrument might not be correct for the particular patient population, the analysis hasn’t been thought through, there’s no hypothesis or objectives listed in the study protocol.”* ***PM-RE13*** |
|  | Lack of adherence to PRO protocol |  |  | *✓* |  |  |  |  |
|  | Inclusion of PRO data as secondary endpoint |  |  | *✓* | *✓* |  |  | *“[…] We don’t see very good advanced planning to use PRO in the drug development process; […] patients reported outcome measures are only classed as exploratory endpoints […] then their impact to regulatory decision-making can only be very limited.”* ***PM-RE13*** |
|  | Lack of PRO information from phase I and II trials limits the design of the PRO component in phase III trials | *✓* |  |  |  |  |  | *"[…] we would get sent the protocol right at the end, right before the trial was going to be sent to ethics or sometimes even after they had received ethical approval. We would make suggestions to improve the protocol with respect to PRO’s and then some of the investigators would be reluctant to make those changes because it meant they would have to do any extensive protocol amendment"* ***AT2*** |
|  | Late or not incorporation of PRO experts in the trial protocol stage | *✓* |  |  |  |  | *✓* |  |

**Table 2 - Suboptimal conduct and analysis quotes**

| **Trial stage** | **Barriers** | **Discussed by** | | | | | | **Illustrative quotes** |
| --- | --- | --- | --- | --- | --- | --- | --- | --- |
|  |  | Academic trialists | Industry trialists | Journal editors | Clinicians | Funders | Policy-makers and regulators |  |
| **Conduct and analysis** | High rates of missing PRO data | *✓* |  |  |  | *✓* | *✓* | *"So if there is a high number of missing values, then the impact is very intensively lowered […]"* ***PM-RE12*** |
|  | Difficulty collecting PRO data among global trials |  | *✓* |  |  |  |  | *“Global trials that involve many countries and languages raise special challenges for PRO where translations are required and collection of PRO may be more challenging to get sites to collect correctly.”* ***IT11*** |
|  | Patient and staff burden | *✓* | *✓* |  |  |  |  | *“The burden on patients is still an issue and a barrier at times. I think we’ve been ineffective in trying to develop parsimonious PRO’s.”* ***PM-RE6*** |
|  | Lack of training for clinicians |  | *✓* |  |  |  |  | *"Physicians don’t have training in patients reported outcomes unless they have an interest in that area."* ***IT4*** |
|  | Lack of expert reviewers to assess PRO trial results |  | *✓* |  |  |  |  | *"Medical journals often lack sufficient experts who can review PRO results because the researchers and clinicians who are journal reviewers are not knowledgeable about PRO."* ***IT11*** |
|  | Clinicians, patients, patient advocates and policy makers lack of understanding and interpretation of PRO data |  | *✓* | *✓* | *✓* | *✓* |  | *“PRO experts, sometimes assume that the clinicians will understand tables and figures and the interpretation of the clinical trial and I think we know from experience that clinicians don’t always get the message.”* ***IT17*** |

**Table 3 - Poor reporting quality quotes**

| **Trial stage** | **Barriers** | **Discussed by** | | | | | | **Illustrative quotes** |
| --- | --- | --- | --- | --- | --- | --- | --- | --- |
|  |  | Academic trialists | Industry trialists | Journal editors | Clinicians | Funders | Policy-makers and regulators |  |
| **Reporting** | **Lack of discussion of PRO outcomes** |  |  |  |  | *✓* |  | *“I think not being published is a big one but also because in the main trial publication, if they are reported then it might just be very minimal information that’s not really…not that it’s not informative, I mean it’s good to know if there are differences between the groups but I think is so much more rich than just that.”* ***AT2*** |
|  | **Lack of inclusion or detailed PRO information within the main trial publication** | *✓* |  |  | *✓* |  |  |  |
|  | **Lack of PRO data explanation in view of other clinical endpoints** | *✓* |  |  | *✓* |  |  |  |
|  | **Lack of journals endorsement** | *✓* |  |  |  |  |  | *“I’m not sure that scientific papers have really got their head around the importance of patient reported outcomes.”* ***FU20*** |
|  | **PRO trial data are never published or it is published years after the main trial publication in a low impact journal** |  |  |  | *✓* | *✓* |  | *“I came across a few trials where the PRO results hadn’t been published […] I saw that certain trials had PRO of secondary endpoint but then when I found the publication that related to it, that was just completely missing and sometimes they would say that the PRO results would be published later but it had been several years down the track.”* ***AT2*** |
|  | **PRO publications in journals report PRO findings in a technically correct language but difficult to understand for patients, advocacy groups and clinicians** |  | *✓* |  |  |  |  | *“There is certainly information that patients want to know about when making a treatment decision and the difficulty is translating those outcomes from the trials into ways that clinicians can understand those outcomes.”* ***IT17*** |
|  | **Restricted access to PRO publications (paywall restrictions)** | *✓* | *✓* | *✓* |  |  |  | *"If you publish it in a very scientific journal and if you are not based in a university, you cannot even read that paper because you don’t have access to those papers. How would you know?"* ***AT3*** |

**Table 4 - Dissemination and uptake of PRO results quotes**

| **Trial stage** | **Barriers** | **Discussed by** | | | | | | **Illustrative quotes** |
| --- | --- | --- | --- | --- | --- | --- | --- | --- |
|  |  | Academic trialists | Industry trialists | Journal editors | Clinicians | Funders | Policy-makers and regulators |  |
| **Dissemination and uptake of results** | **Lack of awareness of PRO data importance among clinicians, researchers, journal editors and sponsors** | *✓* | *✓* | *✓* | *✓* | *✓* | *✓* | *"I think there is still a challenge in the scientific community to see the value of patient reported outcomes as just an important and integral part of our understanding of medicine and medical care and new treatment evaluation".* ***IT11*** |
|  | **Researchers and funders prioritise clinical outcomes rather than PRO data** |  |  | *✓* | *✓* | *✓* |  | *“The main reason is that the high impact journals want survival data and if they’ve got a survival advantage they don’t bother with the quality of life data. […] There’s a study of a drug which has a two-month survival advantage, worse toxicity, quality of life data collected but not published. It’s outrageous.”* ***FU19*** |
|  | **Lack of engagement between academic researchers and research companies with patients to understand patient priorities** |  | *✓* | *✓* | *✓* | *✓* |  | *“The EMA does not permit direct communication from pharma companies to patients, so there are significant challenges sharing more patient-friendly explanations of our PRO research in newsletters, websites, or white papers that could be accessed by patients or clinicians other than with those who participated in the trial or through the Layperson Summary of the trial.”* ***IT11*** |
|  | **Lack of collaboration between PRO researchers within same health research areas** |  |  |  |  |  | *✓* | *"One problem is everyone tries to set up their own shop and so instead of trying to say, we’re going to develop a great tool for gastrointestinal distress, for example, you have six or seven groups that develop groups for very specific disease areas but they all essentially ask the same questions.”* ***PM-RE6*** |
| **Trial stage** | **Barriers** | **Discussed by** | | | | | | **Illustrative quotes** |
|  |  | Academic trialists | Industry trialists | Journal editors | Clinicians | Funders | Policy-makers and regulators |  |
| **Dissemination and uptake of results** | **Lack of law or regulation in the UK to enforce the collection of HRQL in clinical trials** |  |  |  |  |  | *✓* | *"So we are law enforcers if you like. Now, that doesn’t really incorporate PRO’s, there is no specific law if you like, that they’re going to break if they don’t include a PRO or include it in the wrong context or what have you. So it’s almost, I suppose, supplementary information. It’s not regulated in any way in terms of black and white text."* ***PM-RE8*** |
|  | **PROs not automatically becoming health utilities** |  |  |  |  |  | *✓* | *"[…] [PROs] don’t suffer from the problem of not collecting things that will be affected by the disease; they do suffer from the problem that they don’t automatically become utilities, although many of them can be mapped."* ***PM-RE14*** |
|  | **EMA and FDA different perspectives surrounding the inclusion of PROs in clinical trials** | *✓* | *✓* |  |  |  |  | *"[…] the EMA seems to be more willing to accept scientific publications and information in the literature than the FDA is, depending on the reviewing decision, it may be easier or harder to get a PRO in the label, so it’s not an even playing field across the different divisions."* ***IT17*** |
|  | **Difficulty getting funding for PRO research** |  |  |  | *✓* | *✓* |  | *"One of the major funders of research in this country is very unlikely to fund research that has a PROM as a primary outcome. They’ve made that a strategic intent, so the playing field is already biased against PROM’s based research."* ***FU18*** |
