# Supplementary material for: The impact of patient-reported outcome data from clinical trials: perspectives from international stakeholders
Source: J Patient Rep Outcomes. 2020 Jul 2;4:51. doi: 10.1186/s41687-020-00219-4 (PMC7332593; doi:10.1186/s41687-020-00219-4)
Supplement: Supplementary file 5 — Additional file 5: Appendix 5. [file 41687_2020_219_MOESM5_ESM.docx]

**Appendix 5**

**Table 1 - Improved PRO trial design quotes**

| **Trial stage** | **Facilitators** | **Discussed by** | | | | | | **Illustrative quotes** |
| --- | --- | --- | --- | --- | --- | --- | --- | --- |
|  |  | **Academic trialists** | **Industry trialists** | **Journal editors** | **Clinicians** | **Funders** | **Policy-makers and regulators** |  |
| **Design** | **Clear and detailed PRO protocol** |  |  |  |  |  | *✓* | *"The protocol is very complete, very detailed about how to interpret the metrics, how to measure them and standardise their use and so on. The findings are more likely to be considered to be valid and reliable."* ***JE7*** |
|  | **Endorsement of PRO data as key endpoint in clinical trials** |  |  |  |  |  | *✓* | *"I think that they have to be key endpoints in clinical trials […] if you look at arthritis where the main endpoint is a symptom, people talk about symptoms more. I think that when you get into diseases where there are other endpoints, like survival, people tend to focus more on those."* ***CL1*** |
|  | **Early incorporation of a PRO expert in the trial team** |  | *✓* |  |  |  |  | *"There should be a PRO expert on the clinical trial team and at the earliest possible if you start thinking about your PROs at the reporting stage it is far too late. You need to be thinking much earlier on."****AT3*** |
|  | **Adherence to PRO guidelines** |  | *✓* |  |  |  |  | *"[...] the SPIRIT PRO guidelines, the CONSORT guidelines, the guidelines coming out of SISAQOL and ISOQOL, the guidelines that have been recommended by the US FDA about what things should be included when you’re trying to measure well the patients experience."* ***IT11*** |
|  | **Inclusion of patients and clinicians in the trial design stage** | *✓* |  |  |  | *✓* | *✓* | *"It’s really important to speak to clinicians and patients about what issues seem to be most troublesome for patients before they start treatment and then to talk about whether the clinician expects the therapy understudy will have any impact on those particular issues.”* ***AT2*** |

| **Trial stage** | **Facilitators** | **Discussed by** | | | | | | **Illustrative quotes** |
| --- | --- | --- | --- | --- | --- | --- | --- | --- |
|  |  | **Academic trialists** | **Industry trialists** | **Journal editors** | **Clinicians** | **Funders** | **Policy-makers and regulators** |  |
| **Design** | **Regular meetings with regulatory agencies during the planning period and at the end of the trial** | *✓* |  |  |  |  | *✓* | *"We give comments during the planning period so that there are relatively few questions at the end because we will reanalyse the data and go through everything at the end but that’s too late if there is a problem."* ***PM-RE6*** |
|  | **Development of PRO measures while considering health utilities for HTA use** |  |  |  |  |  | *✓* | *"The patient reported outcomes world could think of utilities at the same time as developing their PRO’s. So any patient reported outcome that has got a utility mapping attached to it is very useful."* ***PM-RE14*** |

**Table 2 - Optimal conduct and analysis quotes**

| **Trial stage** | **Facilitators** | **Discussed by** | | | | | | **Illustrative quotes** |
| --- | --- | --- | --- | --- | --- | --- | --- | --- |
|  |  | **Academic trialists** | **Industry trialists** | **Journal editors** | **Clinicians** | **Funders** | **Policy-makers and regulators** |  |
| **Conduct and analysis** | **High completion rates** |  |  |  |  |  | *✓* | *"Try to collect the data from the start until the end of the trial. Also for patients withdrawn from treatment, and also from patients withdrawing from the trials, so there’s a possibility to get an observation from these."* ***PM-RE12*** |
|  | **Training sites on the administration and explanation of PROs** |  | *✓* |  |  |  |  | *"So you have to train the sites and the sites have to be comfortable and understand the tools so they can appropriately explain them to patients."* ***IT5*** |
|  | **Standardisation of PRO tools among therapeutic areas to improve analysis** |  | *✓* |  |  |  |  | *"Having standardised tools across trials helps us understand the trial results and be able to compare things more easily. [...] Certainly in the US with qualification process for PROs, there’s a hope that each of us will not go out and create one off our own tool, instead have some standardisation."* ***IT5*** |
|  | **Make PRO data more readily understandable** | *✓* |  |  |  | *✓* | *✓* | *"One thing is to do a little bit of interpretation to clinicians, so they can look at it and they don’t have to know that a 55 is bad.”* ***IT17*** |

**Table 3 - Improved reporting quotes**

| **Trial stage** | **Facilitators** | **Discussed by** | | | | | | **Illustrative quotes** |
| --- | --- | --- | --- | --- | --- | --- | --- | --- |
|  |  | **Academic trialists** | **Industry trialists** | **Journal editors** | **Clinicians** | **Funders** | **Policy-makers and regulators** |  |
| **Reporting** | **Open access publications** | *✓* |  | *✓* | *✓* |  |  | *“No paywall, it has to be available. There’s no point having a journal that’s accessible to patients if they have to pay for it."* ***JE15*** |
|  | **PRO trial data reported in the main publication and in a high impact journal** | *✓* |  | *✓* | *✓* | *✓* |  | *"The studies which have been impactful have been ones where the quality of life data and the survival data has been published together in a high impact journal"* ***FU19*** |
|  | **Inclusion of a simple English summary of the trial results for use of patients** |  |  | *✓* |  |  |  | *"We insist on having a simple English summary of each paper and when authors submit to us, if the simple English summary isn’t clear, then we won’t read the paper. It goes back to the authors."* ***JE15*** |
|  | **Availability of more journals to publish PRO trial data** | *✓* |  |  |  |  |  | *"So having more PRO clinical trial venues to publish clinical trial results specific for PRO’s would be a good thing […]"****AT10*** |
|  | **Make PRO instruments available through publications** | *✓* |  |  |  |  |  | *“It is extremely important that we make tools publicly available […] and then sharing that information through publication to continue to act at the weight of evidence around the validity of the tool. […] sometimes the PRO becomes part of the company’s intellectual property.”* ***IT5*** |

**Table 4 - Dissemination and uptake of PRO results quotes**

| **Trial stage** | **Facilitators** | **Discussed by** | | | | | | **Illustrative quotes** |
| --- | --- | --- | --- | --- | --- | --- | --- | --- |
|  |  | **Academic trialists** | **Industry trialists** | **Journal editors** | **Clinicians** | **Funders** | **Policy-makers and regulators** |  |
| **Dissemination and uptake of results** | **Adequate funding** | *✓* | *✓* |  | *✓* | *✓* |  | *“I think as a funder we have an opportunity to assert some leverage about the outcomes that go into the trials that are selected for the trials and if you want our funding then you have to meaningfully incorporate PRO’s into the design. You wouldn’t want to skip right into practice without some reasonable understanding about what the PRO’s can predict, how they can affect other treatment outcomes and research itself on whether patients find them meaningful.”* ***FU22*** |
|  | **Funders should clearly express their position about PROs and what they expect from the funded PRO research** |  |  |  | *✓* | *✓* |  |  |
|  | **Funders should require an implementation plan in terms of usage and impact of the PRO clinical trial as a direct requirement of funding** | *✓* |  | *✓* | *✓* |  | *✓* | *“I think funders have a role because they can stipulate, for example, that the work they fund must have some sort of an implementation plan so that work isn’t just completed and then perhaps published in a journal and then never heard from again. Having emphasis on ensuring that there is some pull through into use and impact as a direct requirement of funding would go a long way as well to help the problems.”* ***FU21*** |
|  | **Training courses for clinicians and drug developers** | *✓* | *✓* |  |  |  |  | *“Having training courses that meet the needs of people, nothing too long, targeting events that are already up and running, like having a PRO session at a conference that’s already established rather than having a training day that stands alone.”****AT2*** |

| **Trial stage** | **Facilitators** | **Discussed by** | | | | | | **Illustrative quotes** |
| --- | --- | --- | --- | --- | --- | --- | --- | --- |
|  |  | **Academic trialists** | **Industry trialists** | **Journal editors** | **Clinicians** | **Funders** | **Policy-makers and regulators** |  |
| **Dissemination and uptake of results** | **Communicate PRO research widely through the involvement of key opinion leaders, specifically at healthcare conferences** | *✓* |  |  | *✓* | *✓* |  | *"To allow organisations like the NCRI, ASCO, ESTRO, the organisations that host large healthcare provider conferences to make PROM’s based research a future of their sessions and their main talks and also to improve quality of science communications so that we have skilled science communicators disseminating these results."* ***FU18*** |
|  | **Empowerment of patients through their involvement in discussions and dissemination of PRO trial results** |  |  | *✓* | *✓* | *✓* |  | *"Let’s have a patient standing up saying, my life is better because of this. It’s not just got rid of my cancer, but actually I can cope with life, it’s given me these things to cope with but actually I can cope with those."* ***JE15*** |
|  | **Endorsement of PRO trial studies by key societies to disseminate PRO results and influence healthcare policy** | *✓* |  |  |  | *✓* |  | *"[...] like the charities, like Macmillan, like Cancer Research UK to be having conversations with government and NHS England to make sure that policy is changed. The biggest changes in healthcare don’t come from individuals and from research, they actually come from policy."* ***FU18*** |
